# Proteomic and secretomic comparison of young and aged dermal fibroblasts highlights cytoskeleton as a key component during aging

**DOI:** 10.18632/aging.206055

**Published:** 2024-08-27

**Authors:** Françoise Boismal, Sandy Peltier, Sophie Ly ka so, Guillaume Chevreux, Loïse Blondel, Kévin Serror, Niclas Setterblab, Elina Zuelgaray, David Boccara, Maurice Mimoun, Christelle Guere, Armand Benssussan, Marie Dorr, Gallic Beauchef, Katell Vie, Laurence Michel

**Affiliations:** 1 INSERM UMR_S 976, Skin Research Center, Saint-Louis Hospital, Paris, France; 2 Paris University, Paris Cité, Paris, France; 3 Jacques-Monod Institute, Paris, France; 4 Department of Reconstructive and Plastic Surgery, Saint-Louis Hospital, Paris, France; 5 Technological Platform IRSL, Saint-Louis Hospital, Paris, France; 6 Dermatology Department, Saint-Louis Hospital, Paris, France; 7 Clarins Laboratories, Pontoise, France

**Keywords:** aging, dermal fibroblasts, proteome/secretome, cytoskeleton, wound healing

## Abstract

Crucial for skin homeostasis, synthesis and degradation of extracellular matrix components are orchestrated by dermal fibroblasts. During aging, alterations of component expression, such as collagens and enzymes, lead to reduction of the mechanical cutaneous tension and defects of skin wound healing. The aim of this study was to better understand the molecular alterations underwent by fibroblasts during aging by comparing secretomic and proteomic signatures of fibroblasts from young (<35years) and aged (>55years) skin donors, in quiescence or TGF-stimulated conditions, using HLPC/MS. The comparison of the secretome from young and aged fibroblasts revealed that 16 proteins in resting condition, and 11 proteins after a 24h-lasting TGF-β1-treatment, were expressed in significant different ways between the two cell groups (fold change>2, *p-value* <0.05), with a 77% decrease in the number of secreted proteins in aged cells. Proteome comparison between young and aged fibroblasts identified a significant change of 63 proteins in resting condition, and 73 proteins in TGF-β1-stimulated condition, with a 67% increase in the number of proteins in aged fibroblasts. The majority of the differentially-expressed molecules belongs to the cytoskeleton-associated proteins and aging was characterized by an increase in Coronin 1C (CORO1C), and Filamin B (FLNB) expression in fibroblasts together with a decrease in Cofilin (CFL1), and Actin alpha cardiac muscle 1 (ACTC1) detection in aged cells, these proteins being involved in actin-filament polymerization and sharing co-activity in cell motility. Our present data reinforce knowledge about an age-related alteration in the synthesis of major proteins linked to the migratory and contractile functions of dermal human fibroblasts.

## INTRODUCTION

Skin aging is a genetically-programmed physiological process, dependent on both environment and genetic background [1, 2]. Two combined aging processes co-exist: extrinsic and intrinsic [3, 4]. Extrinsic aging results from exposure to several external factors such as environmental pollution or ultraviolet (UV) radiation among others [5–9], the “exposome” defining all the external exposures to which an individual is subjected from conception to death [8]. Concerning environmental factors, UV contributes to extrinsic skin aging, especially premature aging, up to 80% [9], with several characteristics, including wrinkles and elasticity loss, that are linked to the alteration of extracellular matrix (ECM) components [10, 11]. In contrast, intrinsic skin aging concerns genetic factors, is linked to senescence, [12] and changes in the endocrine environment that reflect the processes of degradation of the whole organism [13, 14].

Dermal fibroblasts dispersed within the dermis are the main actors of sustained ECM renewal by producing collagen and elastin in particular [15, 16]. In case of a cutaneous lesion, fibroblasts secrete matrix metalloproteinases (MMP) that are endopeptidases able to degrade the damaged matrix, including collagenases such as MMP-1 or stromelysins like MMP3, and that are regulated by the specific endogenous tissue inhibitors of metalloproteinases (TIMPs). Fibroblasts will then synthesize a new remodeling matrix and release cytokines to attract macrophages to encompass and degrade debris. They secrete growth factors to stimulate keratinocyte proliferation and migration, leading to complete wound healing [17, 18]. This is the case of young healthy skin, which exhibits as major morphological features, a thick connective tissue maintained by an effective ECM renewal, a well-stratified epidermis, and an effective wound healing process [19, 20]. In contrast, “aged” skin should be sheerer, with a flat epidermis and a loss of ECM leading to a thinner dermis [21, 22]. The ECM morphological changes of the dermis have been shown to be largely responsible for the loss of elasticity and wrinkles as major hallmarks of aging skin [23]. Indeed, dermal fibroblasts undergo physiological changes with age [18, 24], including a permanent secretion of a secretory pole of factors denoted ‘skin aging–associated secreted proteins’ (SAASP) [11], in contrast to young ones which only transiently secrete such factors, for instance during wound healing [25]. Moreover, the decrease in the number of dermal fibroblasts [19] associated with the alteration of the ECM production and the decrease of their migratory capacity with aging [26], as shown recently in a study of comprehensive proteomic database of soluble proteins and exosomal cargo ‘senescence-associated secretory phenotype’ (SASP) factors originating from multiple senescence inducers and cell types including fibroblasts [27] might be involved in the perturbation of wound healing processes observed in the elderly population [3]. The SASP consists of a myriad of cytokines, including chemokines (CXCLs) and growth factors, as well as proteases that all together initiate inflammation, wound healing, and growth responses in nearby cells.

This study aimed to improve knowledge about age-associated molecular alterations in primary cultured fibroblasts from donors older than 55 years (“old fibroblasts”) compared with fibroblasts from donors of less than 35 years (“young fibroblasts”), either in quiescence condition (absence of serum) or under treatment with transforming growth factor-β_1_ (TGF-β_1_) well-known as a major factor for stimulation of wound healing and ECM production by fibroblasts [27]. The secretome and proteome from both aged and young fibroblasts were analyzed by High Pressure Liquid Chromatography/Mass Spectrometry (HPLC/MS). Our comparative analyses of the secretome from young and old fibroblasts revealed a significantly altered expression of less than 75 ones in either quiescence or TGF-β_1_-stimulated condition (fold change>2, *p-value*<0.05). The proteome comparison depicted a significant decrease in fibroblast protein secretion with age and conversely an enhancement of more than 60% of protein cytoplasmic accumulation. This enables us to define the specific role in skin aging of some differentially-expressed proteins, especially those linked with the actin cytoskeleton dynamics. The functions of these proteins were also more deeply analyzed by collagen contraction or cell migration using siRNA transfections.

## MATERIALS AND METHODS

### Fibroblast culture

Dermal fibroblasts were obtained from healthy sun-protected skin of either young (<35 years-old) or aged (>55 years-old) healthy women undergoing breast reduction mammoplasty in our Department of Plastic Surgery, Saint-Louis Hospital. Dermal fibroblasts were cultured as previously described [18] in Roswell Park Memorial Institute 1640 (RPMI) supplemented with 10% foetal bovine serum (FBS), 1% penicillin/streptomycin, 2 mM L-Glutamine, 25 μg/mL amphotericin B and 5 μg/mL of plasmocin (complete RPMI medium) and were used at the 3^rd^ passage to avoid any exhaustion-replicative senescence [11, 28].

### Sample preparation for secretome and proteomic assay

Fibroblasts were either stimulated or not stimulated with TGF-β_1_ for 24h. Then supernatants were centrifuged (400g, 8 min, 4°C) to remove pellets, before addition of protease inhibitors (Sigma-Aldrich, USA; P8340). Adherent fibroblasts were washed, scraped with a spatula, and centrifuged (400g, 8 min, 4°C). Pellets were lysed with 80 μL RIPA buffer (Rockland, USA; MB-030-0050) including protease inhibitors (Sigma-Aldrich; P8340). Protein measurement was performed by Bradford assay (Bio-Rad, USA; #500-0006) and cell lysates were kept at -80°C until they underwent direct digestion by trypsin before analysis at Jacques Monod Institute (JMI, Paris, France).

### Sample preparation prior to LC-MS/MS analysis

50 μg of proteins were precipitated with acetone overnight at -20°C. Samples were centrifuged at 11,000 rpm and 4°C, the pellets were resuspended with 100 μL of NH_4_HCO_3_ 25mM, then heated at 95°C for 10 minutes. The samples were digested overnight at 37°C with 0.4μg of trypsin (Promega, Madison, WI, USA) in a 25 mM NH_4_HCO_3_ buffer per sample. Then peptides were desalted using ZipTip μ-C18 Pipette Tips (Pierce Biotechnology, Rockford, IL, USA).

### LC-MS/MS acquisition

MS grade Acetonitrile (ACN), MS grade H2O and MS grade formic acid (FA) were obtained from Thermo Chemical (Waltham, MA, USA). Samples were analyzed by a Q-Exactive Plus coupled to a Nano-LC Proxeon 1000 (Thermo Fisher Scientific, Waltham, MA, USA).

Peptides were loaded with an online preconcentration method and separated by chromatography using a Pepmap-RSLC C18 column (0.75 x 500 mm, 2 μm, 100 Å) from Thermo Fisher Scientific, equilibrated at 50°C and operating at a flow rate of 300 nl/min. Peptides were eluted by a gradient of solvent A (H_2_O, 0.1 % FA) and solvent B (ACN, 0.1% FA), the column was first equilibrated for 5 min with 95 % of A, then B was raised to 35% in 98 min. Finally, the column was washed with 80% B for 20 min and re-equilibrated at 95% A giving a total run time of 120 min. Peptide masses were analyzed in the Orbitrap cell in full ion scan mode, at a resolution of 70,000, a mass range of *m/z* 375-1500 and an AGC target of 3×10^6^. MS/MS were performed in the top 20 mode. Peptides were selected for fragmentation by Higher-energy C-trap Dissociation (HCD) with a Normalized Collisional Energy of 27% and a dynamic exclusion of 60 seconds. Fragment masses were measured in the Orbitrap, at a resolution of 17,500, with an AGC target of 2×10^5^, an isolation window of 1.4 Da and a mass range of *m/z* 200-2000. Monocharged peptides and unassigned charge states were excluded from the MS/MS acquisition. The maximum ion accumulation times were set to 50 ms for MS and 45 ms for MS/MS acquisitions respectively.

### Data analysis

Label-free quantification was done on Progenesis QI for Proteomics (Waters, Milford, MA, USA). Between runs, alignment of peptide features and normalization based on all peptide ions were both performed by the Progenesis software. An MGF peak file exported from Progenesis was processed on Proteome Discoverer 2.1 with the mascot node (Mascot version 2.5.1). MS/MS spectra were searched against the Swissprot protein database release 2017_04 with the Homo sapiens taxonomy and a maximum of 2 missed cleavages. Precursor and fragment mass tolerances were set to 10 ppm and 0.02 Da respectively. The following post-translational modifications were included as variables: Oxidation (M), acetyl (N-term), phosphor (STY). Spectra were filtered using a 1% FDR with the percolator node. Multivariate statistics on protein measurements were performed using the Progenesis software and protein abundance was inferred using the top 3 method. A two-group comparison parametric t-test was used to determine differential proteins between the following groups: quiescent young versus quiescent old and TGFβ-stimulated young versus TGFβ-stimulated old cells. Proteins expressed in significant different ways were determined with fold>2, *p-value*<0.05. A p-value better than 0.05 was used to filter differential significant candidates.

The expression-based heat maps were done using Heatmapper website, following these characteristics: scale type (select direction to scale values) was “row” type, clustering methods (group the data by similar expression levels) was “average Linkage”, and the distance measurement method was “Eucledian”. The schematic molecular pathways were done using the “PathVisio” Software^®^, after GOrilla and Panthere website analysis.

### Molecular biology

RNA extraction, reverse transcription, and quantitative PCR were performed as previously described [17] and primer sequences were purchased from Eurogentec (Seraing, Belgium).

### Western blot (WB)

Fibroblasts were lysed in Tris-Triton lysis buffer supplemented with protease inhibitor cocktail (Sigma-Aldrich; P8340) and centrifuged (20800g, 15 min, 4°C) before protein concentration determination by Bradford assay. Migration of 50 μg of denatured proteins was performed in 10% Bolt Bis-Tris Plus polyacrylamide gel containing sodium dodecyl sulfate, followed by transfer to a nitrocellulose membrane using horizontal gel transfer device iBlot™ 2. Membranes were blocked with TBS1X-5% nonfat milk-0.1% Tween for 1h and incubated overnight at 4°C with specific primary antibodies (Abcam, UK): CORO1C (ab15719, 1/500); CFL1 (ab11062, 1/10000); ARP2/3 (ab49671, 1/2200); FLNB (ab97457, 1/500); ACTC1 (66125-lg, 1/5000). Secondary antibodies (anti-mouse, Bio-Rad 70-65k, 1/2000 or anti-rabbit, ECL-NA1934VS, 1/1000) coupled to horseradish peroxidase were incubated for 1h. Signal was detected using an enhanced chemiluminescence detection kit (Bio-Rad; #170-5061) and ImageQuant chemiluminescent. Blot quantification was performed using ImageJ software.

**Table:** 

**Gene name**	**Sequence**	
	**Forward**	**Reverse**
**β_2_-microglobulin (β_2_M)**	5’-TGCTGTCTCCATGTTTGATGTATCT-3’	5’-TCTCTGCTCCCCACCTCTAAGT-3’
**Coronin 1C (CORO1C)**	5’-AGGAGCAAGACCCATGAGAG-3’	5’-TTGGTTCCTGCATATTTTTCG-3’
**Cofilin 1 (CFL1)**	5’-AGCCTGCTGGAACCATCTT-3’	5’-ACATCTATGGCCGATGTGG-3’
**Filamin B (FLNB)**	5’-AACAGCCCCTTCACTGTCAT-3’	5’- CATTTACCGGTGCCTCCTC-3’
**Actin Alpha Cardiac Muscle 1 (ACTC1)**	5’- AACCTGGTATTGCTGATCGT-3’	5’- GCTCAGGGGGAGCAATAATC-3’

### Immunofluorescence

Immunostaining was performed on fibroblasts seeded on culture chamber slides. Briefly, fibroblasts were seeded at 3500 cells/well on chamber slide, deprived in 0.5% FBS-containing RPMI medium overnight and then activated with 5ng/ml of TGF-β for 24 hours. Fibroblasts were fixed with 4% paraformaldehyde, then permeabilized with Triton X100-0.1%. Staining was performed by overnight incubation with primary antibodies: CORO1C (ab15719, 1/200); CFL1 (SAB2702206, 1/200); FLNB (ab97457, 1/100); ACTC1 (66125-lg, 1/50), followed by an incubation with AF594 secondary antibodies (anti-mouse, A-11001, 1/2000 or anti-goat, A-11058, 1/1000). Imaging was assessed using an Axiovert 200M-fluo microscope and quantification was processed on ImageJ analysis software.

### Wound closure by collective migration

The IncuCyte^®^S3 Live-Cell Analysis System was used for quantitative label-free collective migration measurements over a 5 day-long lasting culture. Fibroblasts were seeded at 10000 cells per well into 96-well plate to obtain a whole confluency after 24h of culture in complete medium. For siRNA transfection, fibroblasts were transfected with Dharmafect-siRNA mixtures for 5h before cell scratching. For inhibiting the part of cell proliferation during fibroblast migration, mitomycin C (10 μg/ml) was added for 2h before cell scratching. Plates were scratched with a semi-automatized scratch system, creating reproductive regular wounds in each well. Cells were washed to remove cellular debris and complete RPMI medium was added. Plates were placed into the IncuCyte ZOOM^®^ system for scanning every hour until the scratch wound was completely closed. Captured images were analysed using the integrated software by calculating the cell confluence in the wound.

### Collagen gel (lattice) contraction assay

Fibroblasts (100000 cells/mL) were added to a solution containing Type I Collagen (Corning™), 0.006N acetic acid, 0.005N NaOH and incubated in a 6-well plate for 1 h at 37°C to induce gelation. Two lattices were made for each condition. Dermal collagen lattices were detached allowing floatation. OPTIMEM 1% FBS was added at 2X concentration and pictures were taken daily for one week. The lattice area was measured twice by ImageJ.

### Transfection assay

Fibroblast siRNA transfection was carried out using Lipofectamine 2000 Transfection Reagent (Life Technologies, USA) according to the manufacturer’s protocol. Briefly, Lipofectamine was diluted in OPTIMEM medium (1/25) as well as the siRNA stock solution (1/50) and incubated for 5 min at RT. Lipofectamine mix was added to the siRNA mix and incubated for 20 min at RT. The resultant mix was then diluted with OPTIMEM to reach a final concentration of 100 nM of siRNA. Cells were transfected with 100 nM of siRNA targeting p16 (Thermo Fisher Scientific) for 24h before RNA and protein extraction. As control siRNA (SiControl), an irrelevant siRNA sequence pool (Thermo Fisher Scientific) was used.

### Statistical analysis

Results were expressed as mean ± SEM from independent experiments. Data were symmetrically distributed in a normal distribution. One Way ANOVA Multiple comparisons or Wilcoxon Rank-Sum test were performed using Prism^®^ (GraphPad 9.1.1) analysis. Differences were considered as significant at *: *p*<0.05; **: *p*<0.01; ***: *p*<0.001.

## RESULTS

### Secretome study reveals a decrease in protein secretion with age

**Figure 1:**
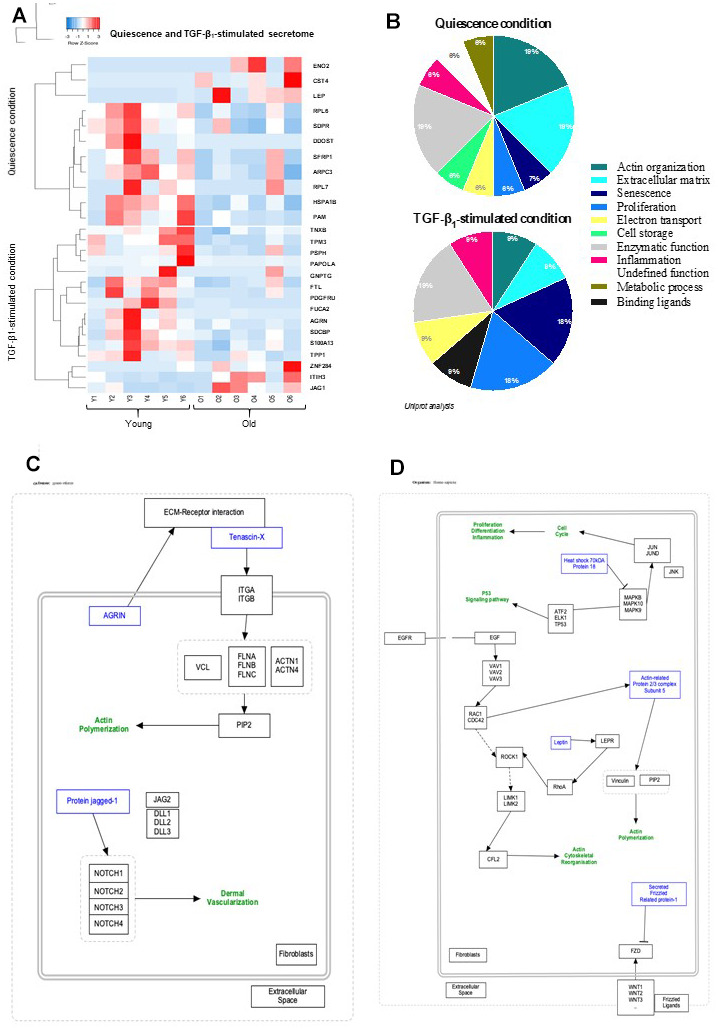
**Quiescence and TGF-β_1_-stimulated SECRETOME heat-map and schematic representations of molecular pathways.** (**A**) Heat-maps of differentially-expressed proteins in young and old fibroblasts (n=6) (HeatMapper). (**B**) Analysis of cellular processes linked to differentially-expressed proteins (GOrilla and Panthere). (**C**) Representation of signaling pathways and links between differently expressed proteins in quiescence and (**D**) TGF-β_1_-treated condition (PathVisio).

Senescent cells—and more importantly SASP, the factors they secrete—are widely accepted as drivers of skin aging and multiple age-related cutaneous pathologies. In the present analysis of fibroblast secretome, a total of 945 different proteins between young and old cells was detected in quiescence condition with a significant differential expression of only 16 proteins according to the chosen criteria of selection: fold change>2, *p*-value<0.05. Under TGF-β_1_ addition, 944 different proteins between young and old fibroblasts were identified by HPLC with a significant differential expression of 11 proteins (fold change>2, *p*-value<0.05). The secretome expression-based heat map (Figure 1A) showed that the differentially-secreted proteins were highly expressed in young fibroblasts compared to those from old ones in either quiescent (13/16) or TGF-β_1_-stimulated (8/13) conditions. The role and function of each identified protein was deeply investigated by a global literature review leading to a general secretory pattern associated with skin aging and correlated with cellular pathways (Figure 1B). We underlined that 19% of the proteins differentially-expressed in quiescence condition between young and old fibroblasts are associated with either actin structure, ECM organization, or enzymatic function, whereas other biological processes including proliferation, inflammation, metabolism, or senescence represent less than 8% each. TGF-β_1_-treatment induced alterations mainly related to enzymatic function (19%), proliferation/senescence (18%), or actin and ECM organization for 9% each (Figure 1B). A schematic representation of proteins linked to a specific pathway was obtained for quiescence and activation conditions (Figure 1C, 1D, respectively). Noteworthy, the organization of actin and ECM structures appeared to be the major categories linked to the differentially-expressed proteins in both secretome and proteome.

### Proteome study revealed a gain in protein production with age

**Figure 2:**
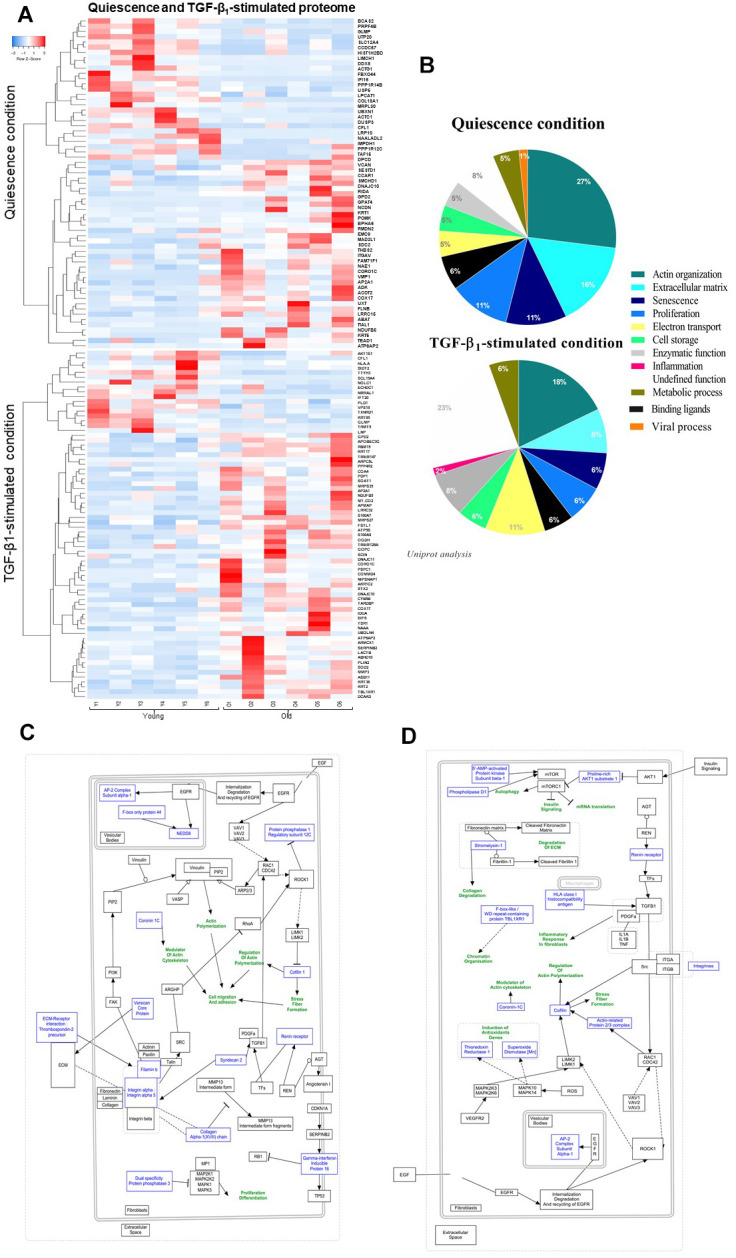
**Quiescence and TGF-β_1_-stimulated PROTEOME heat-map and schematic representations of molecular pathways.** (**A**) Heat-maps of differentially-expressed proteins in young and old fibroblasts (n=6) (HeatMapper). (**B**) Analysis of cellular processes linked to differentially-expressed proteins (GOrilla and Panthere). (**C**) Representation of signaling pathways and links between differentially-expressed proteins in quiescence and (**D**) TGF-β_1_-treated condition (PathVisio).

Proteomic study identified a total number of 4169 proteins between quiescent young and old fibroblasts in quiescence condition from which only 63 proteins were statistically differentially-expressed (fold change>2, *p*-value<0.05). Under TGF-β1 treatment, the young versus old fibroblast comparison of proteomes revealed a difference of 4169 proteins from which only 73 were retained as differentially-expressed according to the criteria of fold change>2 and *p*-value<0.05. As revealed by the proteome expression-based heat map (Figure 2A) most of these proteins were detected in old fibroblasts rather than in young ones, either in quiescent (34/63) or TGF-β1-treatment (56/73) conditions. Similar to the secretome comparison, actin organization/cytoskeleton function represents 27% of the 63 differentially-expressed proteins in old fibroblasts (Figure 2B) and the same fields were detected under TGF-β_1_-treated condition: actin with a 18% rate and ECM organization for 8%, although a 23% group of proteins with undefined functions were detected in old fibroblasts (Figure 2B). The schematic pathway representations (Figure 2C, 2D) reported that most of the proteins were linked to actin-cytoskeleton regulation, inflammation (due to the TGF-β_1_ treatment), and ECM maintenance.

### Actin-related proteins are a key role in fibroblast features during aging

More in depth, we selected four differentially-expressed proteins, either secretome or proteome, based upon their actin-relation and high expression levels (Table 1): Cofilin (CFL1) and Coronin 1C (CORO1C) which are two major proteins involved in actin-filament polymerization and might share co-activity in cell motility; noteworthy, they were detected in both quiescent and TGF-β1-treated proteome condition; Filamin B (FLNB) which is involved in organization of networks of filamentous actin and stress fibers [29, 30] and Actin alpha cardiac muscle 1 (ACTC1), as one of the six actin isoforms.

**Table 1:** Characteristic details of the selected actin-associated proteins chosen for RTqPCR, WB, and IF analysis.

**Name**	**Gene**	**Uniprot ID**	**Fold change (O/Y)**	**Variations (O>Y; or Y>O)**	**Link**
**Cofilin - 1**	*CFL1*	P23528	Quiescence proteome **= 0.30** TGF-β1-stimulated proteome **= 0.23**	Y < O	Actin Fiber Regulation (polymerization/depolymerization)
**Coronin-1C**	*CORO1C*	Q9ULV4	Quiescence proteome **= 35.40** TGF-β1-stimulated proteome **= 3.29**	O > Y	Actin Fiber Regulation (polymerization/depolymerization)
**Filamin B**	*FLNB*	O75369	Quiescence proteome **= 2.07**	O > Y	Link between extracellular matrix and intracellular cytoskeleton
**Actin, alpha cardiac muscle 1**	*ACTC1*	P68032	Quiescence proteome **= 0.01**	Y < O	Actin Cytoskeleton

O = old; Y = young.

**Figure 3:**
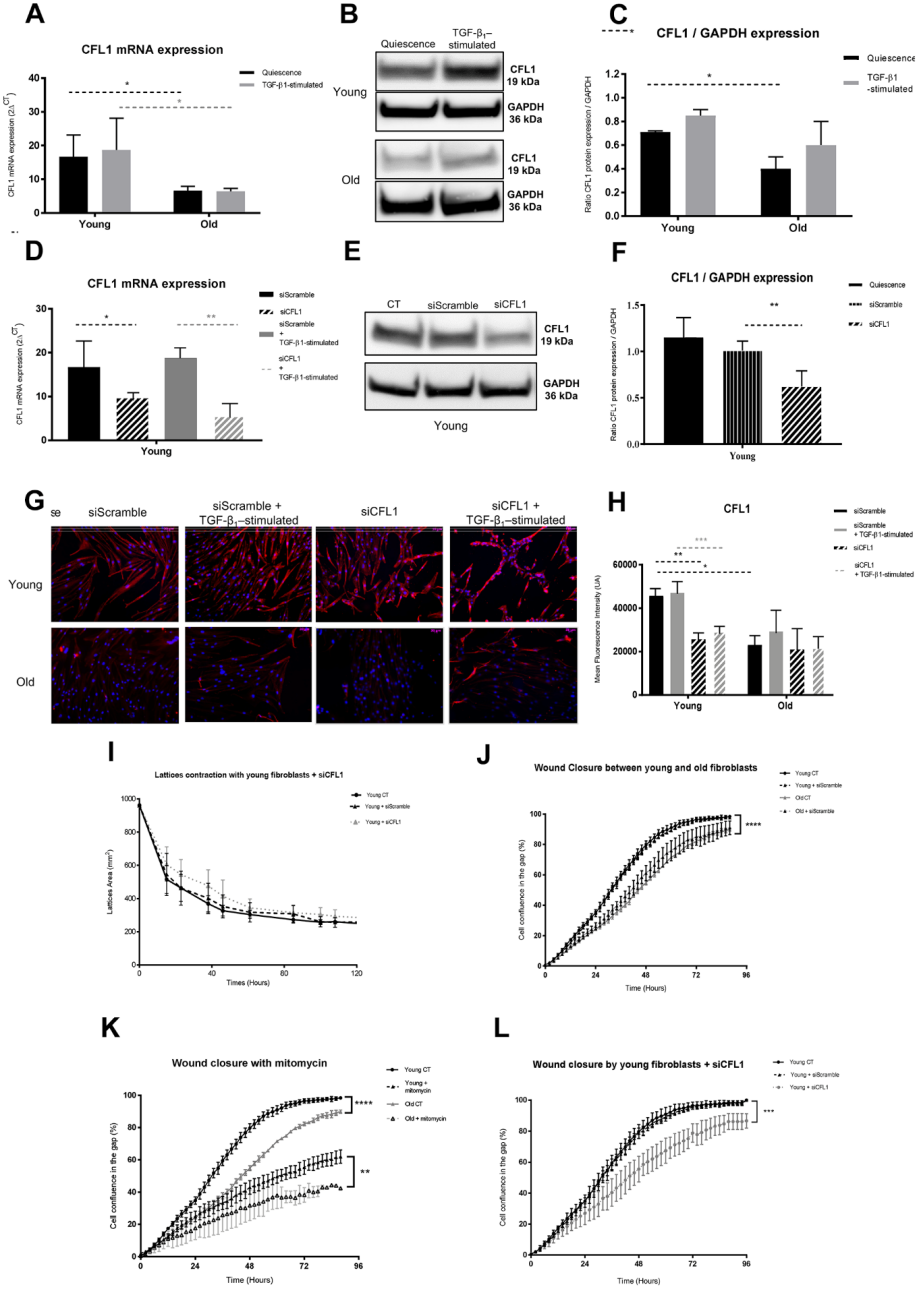
**CFL1 analysis.** (**A**) mRNA CFL1 expression (RT-qPCR) of fibroblasts in quiescence or after 24h of TGF-β-1 stimulation (Mean±SEM, n= 6 for young and old cells); (**B**) one representative western blot of CFL1 and GAPDH expression and (**C**) mean (±SD) quantification of CFL1/GAPDH ratio expression in quiescence or after a 24h-TGF-β-1 stimulation of young or old fibroblasts (n=3 per type); (**D**) mRNA expression quantified by RT-qPCR after transfection with specific siRNA for CFL1 (siCFL1) or with Scramble siRNA (siScramble), in quiescence or after a 24h-TGF-β-1 stimulation (Mean±SD, n=6 per type); (**E**) after transfection of young fibroblasts with specific siCFL1 or with siScramble, one representative western blot of CFL1 and GAPDH expression and (**F**) mean WB quantification of CFL1/GAPDH ratio expression (Mean±SD, n=3); (**G**) one representative immunofluorescence staining after transfection with specific siCFL1 or with siScramble of one representative young and one old fibroblast population, either in quiescence or after a 24h-TGF-β-1 stimulation and (**H**) in the same conditions, mean quantification of immunofluorescence staining from young or old fibroblasts (Mean±SD, n=6); (**I**) collagen gel contraction (lattice) of young fibroblasts either from control cultures or after transfection with specific siCFL1 or with siScramble during 120h (Mean±SD, n=6); (**J**) wound closure over 96h as measured by % of cell confluency for analysis of migration of young control fibroblasts (CT) or after transfection with siCFL1 or siScramble siRNA (Mean ±SD, n=6); (**K**) wound closure over 96h as measured by % of cell confluency for comparison of migration between young and old fibroblasts (Mean ±SD, n=6 per cell type); (**L**) wound closure over 96h as measured by % of cell confluency for analysis of migration with or without mitomycin-C inhibitor of young and old fibroblasts (Mean ±SD, n=6 per cell type). *p*-value *<0.05, **<0.01, ***<0.001.

CFL1 is an actin filament-binding monomer [31]. It is regulated by Actin-related protein 2/3 complex subunit 3 – ARP2/3 (ARPC3) which has been described as a major actin-branched protein [32] and although its abundancy level was twice lower in the proteome of aged fibroblasts as compared to that of young cells, this was without significance. The proteome analysis showed that CFL1 was decreased in old fibroblasts with a fold change (Old/Young) of 0.30 and 0.23 in quiescence and TGF-β_1_-treated condition, respectively (Table 1). This CFL1 decrease with aging was confirmed for mRNA (Figure 3A) and protein expression detected by western blot (WB) and Immunofluorescence (IF) analysis (Figure 3B, 3G). The transfection of CFL1-specific siRNA in young fibroblasts induced an effective down-regulation of CFL1 at mRNA level (42% and 69% in quiescence and stimulated condition, respectively) and protein expression (Figure 3D–3F). However, CFL1 down-regulation did not alter the contractibility capacity of young fibroblasts as shown by gel contraction assay (Figure 3I), whereas it induced some cellular morphological changes, including a compaction of cytoplasm in spindles associated with rounder cell shapes (Figure 3G). CFL1 depletion by siRNA transfection in young fibroblasts also induced a significant decrease in wound closure compared to wild-type young cells (Figure 3H) as shown by using a wound healing-scratching assay in which fibroblasts dynamically migrate to close a linear scratch-induced wound *in vitro*. The present results confirmed our previous results showing that the migration of young fibroblasts was significantly higher than that of old ones (Figure 3K). This significant difference was not due to a higher proliferative capacity of young fibroblasts, since it was still observed in the presence of mitomycin-C used as a well-known proliferation inhibitor (Figure 3I).

**Figure 4:**
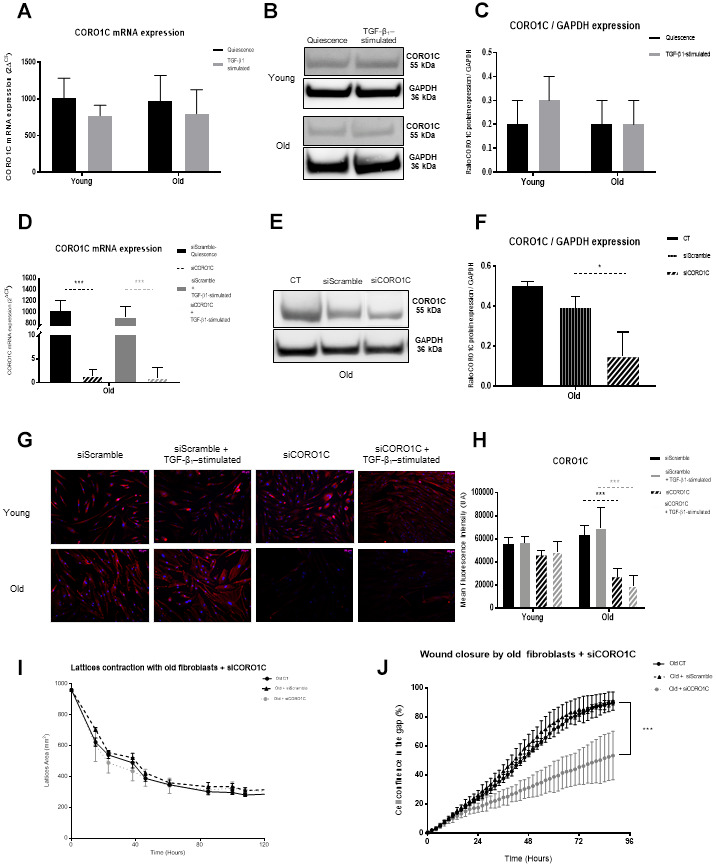
**CORO1C analysis.** (**A**) mRNA CORO1C expression (RT-qPCR) of fibroblasts in quiescence or after 24h of TGF-β-1 stimulation (Mean±SEM, n= 6 for young and old cells); (**B**) one representative western blot of CORO1C and GAPDH expression and (**C**) mean (±SD) quantification of CORO1C/GAPDH ratio expression in quiescence or after a 24h-TGF-β-1 stimulation of young or old fibroblasts (n=3 per type); (**D**) mRNA expression quantified by RT-qPCR after transfection with specific siRNA for CORO1C (siCORO1C) or with Scramble siRNA (siScramble), in quiescence or after a 24h-TGF-β-1 stimulation (Mean±SD, n=6 per type); (**E**) after transfection of young fibroblasts with specific siCORO1C or with siScramble, one representative western blot of CORO1C and GAPDH expression and (**F**) mean WB quantification of CORO1C/GAPDH ratio expression (Mean±SD, n=3); (**G**) one representative immunofluorescence staining after transfection with specific siCORO1C or with siScramble of one representative young and one old fibroblast population, either in quiescence or after a 24h-TGF-β-1 stimulation and (**H**) in the same conditions, mean quantification of immunofluorescence staining from young or old fibroblasts (Mean±SD, n=6); (**I**) collagen gel contraction (lattice) of old fibroblasts either from control cultures or after transfection with specific siCORO1C or with siScramble during 120h (Mean±SD, n=6); (**J**) wound closure over 96h as measured by % of cell confluency for analysis of migration of old controls fibroblasts (CT) or after transfection with siCORO1C or siScramble siRNA (Mean ±SD, n=6). *p*-value *<0.05, **<0.01, ***<0.001. (**J**) Schematic representation of the actin dynamic polymerization between CFL1, CORO1C and ARP2/3, regulators, adapted from *Mol Biol Cell. 2010; 21:3529-39.* Hypothetically, the low level of ARP2/3 in old cells might favor a potent deficiency in actin polymerization with as consequences, alteration of cell motility and capacity to interact with the ECM components. This could be reinforced by the low levels of CFL1. Moreover, the increase of CORO1C in old fibroblasts should induce a higher strength of the actin filaments, leading to cell rigidification with no renewal in the actin complex as a consequence of loss in cell contractility and effective migration.

CORO1C, also designed as coronin-3, is an actin cytoskeleton regulator/modulator active in lamellipodia, protrusion formation and invasion [33]. In contrast to CFL1, CORO1C expression was upregulated in old fibroblasts in both quiescence and TGF-β_1_-activated proteomic condition, with a fold change (Old/Young) of 35.40 and 3.29, respectively (Table 1). However, CORO1C mRNA expression and the protein detected by WB analysis did not show a significant difference between both young and old fibroblasts (Figure 4A–4C). As previously suggested by Waldera-Lupa et al*.* (2014), this absence of significant changes of the corresponding mRNA transcripts might suggest that some age-associated alterations detected at the proteome level are likely caused by other processes, such as post-transcriptional regulation, translation efficiency, protein stability or modifications, rather than by differential regulation of gene expression. This is confirmed by the slight, non-significant data depicted by the immunoblot of old and young cells, TGF-β1-treated or not (Figure 4B). CORO1C expression was significantly depleted by siRNA transfection in old fibroblasts in quiescence and stimulated condition for both mRNA and protein expression, with an inhibition >98% and >60%, respectively (Figure 4D–4F). The CORO1C immunofluorescence analysis confirmed the increased expression in old cells and its depletion after siRNA transfection (Figure 4G, 4H). Although such siRNA-induced depletion of CORO1C did not alter old fibroblast contraction ability in collagen lattices (Figure 4I), it did induce a significant alteration in their migratory capacities (Figure 4J).

CORO1C might act together with CFL1 which could be regulated by the actin-associated protein ARP2/3 complex, ARPC3 or ARP2/3. Noteworthy, a twice lower expression of ARPC3 was detected in the proteome analysis of quiescent old fibroblasts although with no significance and a significant lower level of this protein (fold ratio of 0.48 between old and young cells) was measured in the quiescent secretome (*cf* Figure 1A). The secretion of this key factor in actin filament branching and polymerization was quite surprising and was not detectable in cell supernatants by any method we used. As presented in Supplementary Figure 1, the ARP2/3 mRNA (Supplementary Figure 1A) and protein expression (Supplementary Figure 1B, 1C, 1G, 1H) were not different in young and old fibroblasts, but the efficient ARP2/3 depletion (more than 96%) at mRNA and protein level by siRNA transfection (Supplementary Figure 1E, 1F) did significantly decrease the migration of young fibroblasts in scratching assays (Supplementary Figure 1I).

Altogether, these data lead us to suggest that the three molecules CFL1, CORO 1C and ARP2/3 act together to regulate the actin cytoskeleton and the cytoskeleton regulation itself, as illustrated by the schematic representation of the dynamic actin-polymerization process we propose. This could explain how these molecules alter fibroblast function with aging by acting together for actin organization and consequent ECM disorganization by decreasing/increasing in aged cells (Supplementary Figure 1J). This might induce defective migration during wound healing or dermal defective appearance of aged skin.

**Figure 5:**
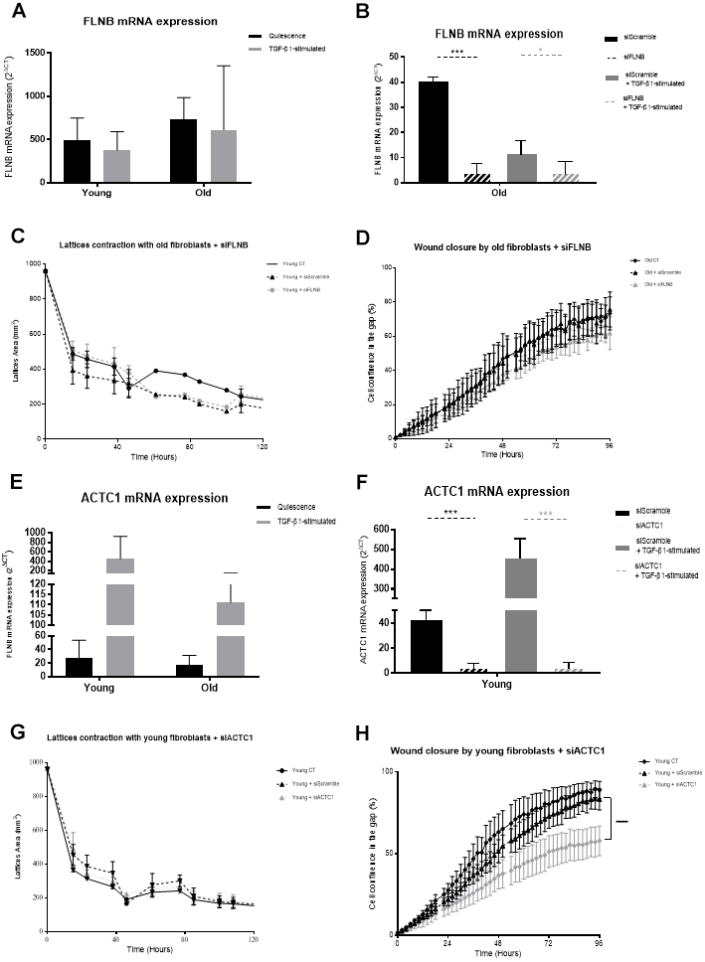
**FLNB and ACTC1 analysis.** (**A**) mRNA FLNB expression (RT-qPCR) of fibroblasts in quiescence or after 24h of TGF-β-1 stimulation (Mean±SEM, n= 6 for young and old cells); (**B**) mRNA expression quantified by RT-qPCR after transfection with specific siRNA for FLNB (siFLNB) or with Scramble siRNA (siScramble), in quiescence or after a 24h-TGF-β-1 stimulation (Mean±SD, n=6 per type); (**C**) Collagen gel contraction (lattice) of old fibroblasts either from control cultures or after transfection with specific siFLNB or with siScramble during 120h (Mean±SD, n=6); (**D**) Wound closure over 96h as measured by % of cell confluency for analysis of migration of old control fibroblasts (CT) or after transfection with FLNB or siScramble (Mean±SD, n=6); (**E**) mRNA ACTC1 expression (RT-qPCR) of fibroblasts in quiescence or after 24h of TGF-β-1 stimulation (Mean±SEM, n= 6 for young and old cells); (**F**) mRNA expression quantified by RT-qPCR after transfection with specific siRNA for ACTC1 (siACTC1) or with Scramble siRNA (siScramble), in quiescence or after a 24h-TGF-β-1 stimulation (Mean±SD, n=6 per type); (**G**) Wound closure over 96h as measured by % of cell confluency for analysis of migration of old control fibroblasts (CT) or after transfection with siACTC1 or siScramble (Mean±SD, n=6); (**H**) Collagen gel contraction (lattice) of young fibroblasts either from control cultures or after transfection with specific siACTC1 or with siScramble during 120h (Mean±SD, n=6); *p*-value *<0.05, **<0.01, ***<0.001.

Besides the actin-associated proteins, FLNB was retained as one isoform of the superfamily of cytoskeletal filamins that organize filamentous actin in networks and stress fibers [29, 30]. FLNB acts as an actin-binding protein that interacts with multiple receptors and intracellular proteins for regulation of cytoskeleton-dependent cell proliferation, differentiation, and migration [34, 35]. According to the proteome of quiescent fibroblasts, an increase of FLNB was observed under aging with a 2.07-fold change (Old/Young) (Table 1). However, no difference of FLNB mRNA levels was measured between young and old fibroblasts, either activated or not by TGF-β1 (Figure 5A). Depletion of FLNB mRNA expression by siRNA (>87%) (Figure 5B) induced a significant decrease in migratory capacities of old fibroblasts (Figure 5C), although it did not alter their contractibility (Figure 5D).

ACTC1 was also chosen as one of the six actin isoforms [36], with actins playing a major role in the shape and cell movement processes [37]. ACTC1 is predominantly localized in the heart and its alteration by methylation has been linked to cardiopathies [38]. The proteome analysis showed a decrease in the expression of ACTC1 in old fibroblasts with a 0.01-fold change (Old/Young) (Table 1). As for FLNB expression, ACTC1 mRNA levels were not significantly higher in young versus old fibroblasts, either activated or not (Figure 5E) although mRNA detection in young cells was downregulated by 83% after siRNA transfection (Figure 5F). Such ACTC1 depletion in young fibroblasts did significantly impact their migratory capacities (Figure 5G), but did not alter their contractibility (Figure 5H).

## DISCUSSION

To identify relevant molecules involved in the main role of human dermal fibroblasts during the process of wound healing and skin aging, a comparative analysis of the secretome and proteome of 12 cultures of dermal fibroblasts, freshly-isolated from young and mature skin, was carried out by HPLC/MS in either quiescence or TGF-β_1_-treated condition, in the absence of senescence by specific inducers, as described in other previously reported aging model systems [11, 27]. It must be noted that the analyses were assessed in the absence of serum in the culture medium, 24h before and during cell stimulation in order to avoid any serum protein contamination for these secretomic and proteomic assays.

Secretome comparison between young and old fibroblasts showed that more than 70% of the differentially-secreted proteins were significantly downregulated in old fibroblasts, and the main proportion of these secreted proteins was linked to the actin cytoskeleton in both quiescence and TGF-β_1_-activated condition, suggesting that aging brings a decrease in fibroblast communication and migration within the environment. Other differentially-secreted proteins belong to several biological processes for less than 8% each, including inflammation, electron transport, cell storage, metabolism or senescence. This is in accordance with previous data about aging, reporting that cells become senescent and exert numerous effects through their secretory products known as SAASP [11, 39], and that this could be involved in wound healing alteration during skin aging [40]. Our data are in accordance with the observation obtained from the “SASP Atlas” published by Basisty et al. (2020) as a comprehensive proteomic database of soluble proteins originating from multiple senescence inducers and cell types, showing that the largest pathway associated with all inducers related to tissue and cell structure, mainly include extracellular matrix organization, actin cytoskeleton, integrin interactions, and peptidase regulation.

In the proteome analysis contrary to the secretome one, more than half of the differentially-expressed proteins were upregulated in old cells, but again, almost 25% of altered proteins exhibited a major link to the actin organization/cytoskeleton in either quiescence or activated conditions. As actin organization in fibroblasts has been shown to be substantial during aging [41], our results suggest that actin-related proteins should favor actin-cytoskeleton binding in aged fibroblasts, leading to their rigidification and loss of intracytoplasmic structural organization with consequent morphological changes and consequent alteration in cell migration and wound healing resolution. This might induce a loss of active contractile actin-stress fibers, resulting in nuclear deformation and physical disruption of the cells that could limit their contractibility potential and cause further contraction of the wound merges by fibroblasts.

This might also disturb the production or degradation of the ECM components which will also limit wound healing, as suggested by [42]. This will alter the structure of the dermis composition in links with ECM modification by fibroblasts, as previously reported [43] and supported by our present functional results. Indeed, the second group of upregulated proteins (around 10%) in old fibroblasts was linked to ECM organization.

As previously reported [18, 26], we confirmed the significantly lower migratory capacities of old fibroblasts compared to young ones. Beside this fibroblast migration deficiency with age, it has been shown that their numbers decreased [19] and the collagen fibers are more or less abundant and often disorganized and fragmented [44], leading to a defective dermal structure in the elderly. Mechanosensation designs the cell responses to any ECM changes, allowing mechanotransduction through biochemical signals thanks to actin cytoskeleton dynamics [45]. With aging, these mechanical features were shown to be impaired [16]. In particular, the ECM disorganization was reported to favor an alteration in the migration of fibroblasts to the wound site [46]. The modification of ECM components we depicted in old fibroblasts could increase such defect of skin repair. This altered dynamics with aging could be related to our present data showing that the significant alteration of actin-associated proteins, such as CFL1, CORO1C and ARP2/3 in old fibroblasts, did impair cell migration and wound healing.

Accordingly, CFL1, which is a key regulator in actin filament assembly/disassembly (ADF/cofilin), acts upon actin levels and relative binding-proteins – a low cofilin/actin ratio would favor a permanent filament turn-over, whereas a high ratio facilitates CFL1 binding to F-actin in order to stabilize it in saturate severing fragments [47]. CFL1 also works together with the ARP2/3 complex to induce Rac/Rho pathway activity. Moreover, CLF1 loss has been shown to favor an uncontrolled accumulation of contractile actin stress fibers, with a consequent increase in intracellular actin-myosin tension which might promote nuclear deformation and physical disruption as suggested by [48]. We observed a decrease in CFL1 mRNA and protein expression in old fibroblasts and it was indeed associated with a less-effective wound closure by collective fibroblast migration.

CORO1C, which belongs to a family of highly conserved actin-binding proteins that bind to filamentous actin/F-actin, acts as a key regulator of actin assembly/dynamics by working with ARP2/3 complex as well as with Rac1 [49]. CORO1C is located in the lamellipodia, evoking a potent role in motility and migratory ability. Indeed, redistribution of Rac1 from the back of the cell to the front was shown to enable migration, revealing the major involvement of cytoskeleton structure in migrating cells [50]. The overexpression of CORO1C in old fibroblasts seems to down-regulate their motility, although its depletion did not allow it to reach the level of migration of young fibroblasts, suggesting that CORO1C alone was not sufficient to affect motility.

ARP2/3 was another actin-related protein that was down-regulated in old fibroblasts. Identified as a profilin-binding protein, ARP2/3 is a key regulator in the cellular cytoskeleton. It has been shown to be involved in the regulation of actin polymerization although it could not activate this polymerization alone [51]. It can be active with an activating nucleation enhancer factor (NPF) on the formation of branched-actin networks [52], forming the F-actin bypass networks [53]. As with CORO1C, ARP2/3 localization in lamellipodia enables fibroblast migration [54]. Our data demonstrated the involvement of this complex in collective migration since ARP2/3 downregulation by siRNA induced a decrease in migratory capacity of young fibroblasts (data not shown), just as CFL1 did. There is a potent link between the three actin-related molecules that might complementary act together, in common with Rac1, in the regulation of the actin cytoskeleton and the cytoskeleton itself, as illustrated in Figure 4K, to schematize their complementary function in the dynamic actin-polymerization process that could be deregulated with aging.

FLNB is another actin-related protein with a key role in the migratory turnover process by connecting filaments of the actin cytoskeleton to the plasma membrane, allowing cells to change shape and to move [55]. Targeting FLNB in fibroblasts was shown to increase matrix metallopeptidase-9 (MMP9) and VEGF secretion which led to induction in tumor growth [56]. Integrin activation has been shown to be involved in the FLNB role in cellular migration [30, 57] in accordance with our data on the decrease of migratory capacity of old fibroblasts after transfection of siRNA targeting FLNB. However, we did not observe a significant alteration in integrin and focal adhesion expression in proteome data or by immunofluorescence analysis, nor an increase in VEGF production by transfected fibroblasts (data not shown).

As actins play a key role in the migratory capacity through their involvement in motility, protusion cell cycle, traction and tail retraction [58, 59], the very low expression of one of the six actin isoforms, ACTC1, in old fibroblasts might be in relation with their altered migratory capacities since ACTC1 depletion in young cells did reduce their migration, suggesting a major involvement of this protein in the wound closure process through fibroblast functions.

Further, the dynamic features, including motility and mechanical forces exhibited by young and old fibroblasts, will be analyzed using Traction Force Microscopy (TFM) and single cell migration. A combined downregulation of the three actin-related proteins we studied would confirm their close association to cytoskeleton behavior and cell motility/migration to further modulate their involvement in aging. Yet they do not seem to be major actors in the merge of traction and contraction of the wounds.

Altogether, our data showed that regulation of fibroblast cytoskeleton through actin-related proteins, combined with ECM organization, is a key process to control skin aging. Noteworthy, our comparative data from the proteomes described several unknown proteins or with undefined functions. These proteins should be further studied in order to investigate more deeply the ongoing aging process and its regulation.

## CONCLUSIONS

This study revealed a significant decrease in fibroblast protein secretion with age and conversely an enhancement of more than 60% of cytoplasmic protein accumulation. Proteins associated with actin and ECM organization revealed to be the two main fields of proteins modified during aging. In depth analysis of actin-related proteins showed evidence of involvement of proteins like CFL1, CORO1C, ARP2/3-complex, FLNB and ACTC1, in cytoskeleton organization and fibroblast migration capacities, offering new targets to slow characteristic features of skin aging.

## Supplementary Material

Supplementary Figure 1
