# Supplementary material for: Proteomic and secretomic comparison of young and aged dermal fibroblasts highlights cytoskeleton as a key component during aging
Source: Aging (Albany NY). 2024 Aug 27;16(16):11776–95. doi: 10.18632/aging.206055 (PMC11386920; doi:10.18632/aging.206055)
Supplement: Supplementary Figure 1 [file aging-16-206055-s001.pdf]

SUPPLEMENTARY FIGURES

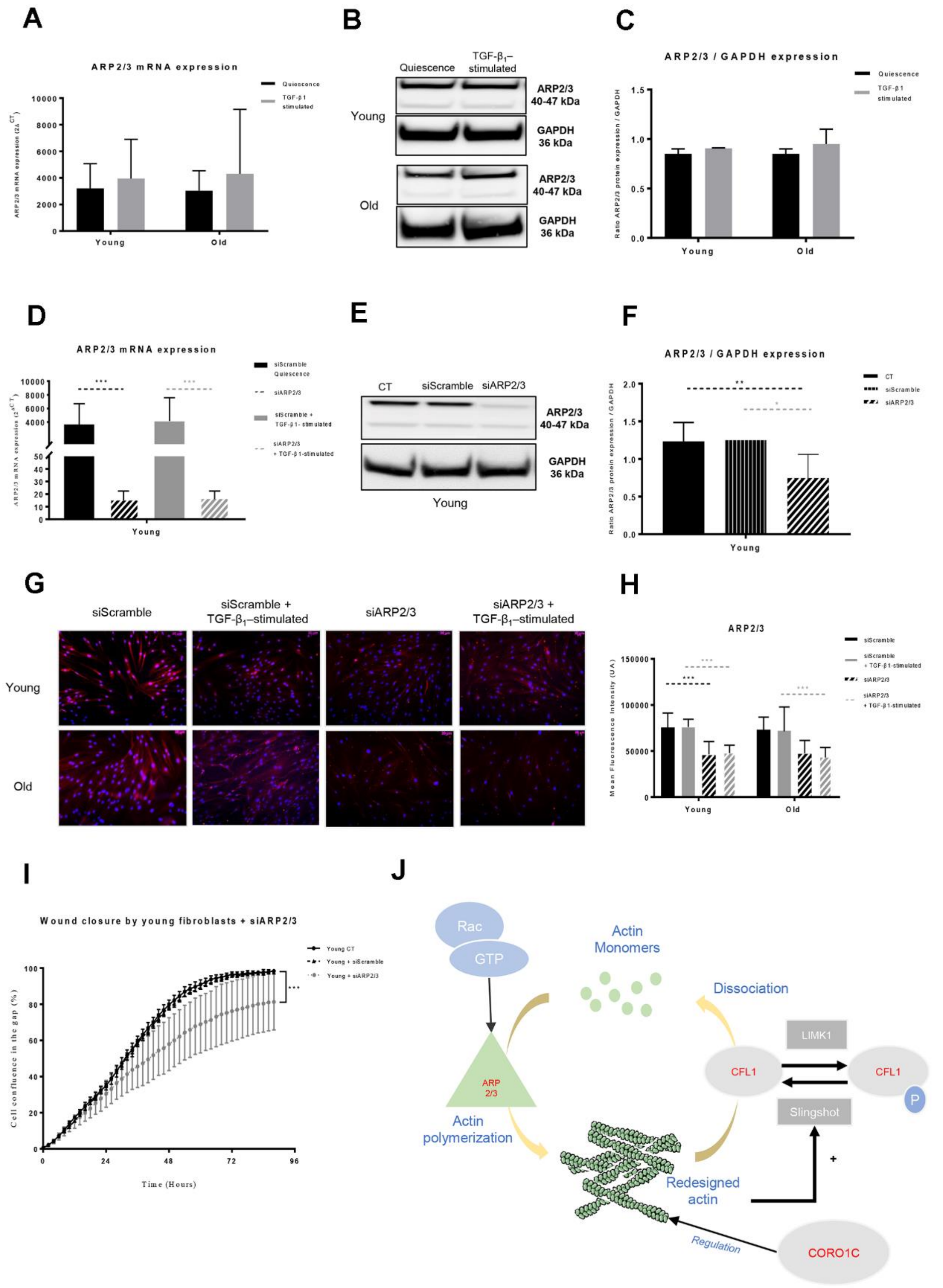

Supplementary Figure 1. ARP2/3 analysis. (**A**) mRNA ARP2/3 expression (RT-qPCR) of fibroblasts in quiescence or after 24h of TGF-β-1 stimulation (Mean±SEM, n= 6 for young and old cells); (**B**) one representative western blot of ARP2/3 and GAPDH expression and (**C**) mean (±SD) quantification of ARP2/3 /GAPDH ratio expression in quiescence or after a 24h-TGF-β-1 stimulation of young or old fibroblasts (n=3

per type); (**D**) mRNA expression quantified by RT-qPCR after transfection with specific siRNA for ARP2/3 (siARP2/3) or with Scramble siRNA (siScramble), in quiescence or after a 24h-TGF-β-1 stimulation (Mean±SD, n=6 per type); (**E**) after transfection of young fibroblasts with specific siARP2/3 or with siScramble, one representative western blot of ARP2/3 and GAPDH expression and (**F**) mean WB quantification of ARP2/3 /GAPDH ratio expression (Mean±SD, n=3); (**G**) one representative immunofluorescence staining after transfection with specific siARP2/3 or with siScramble of one representative young and one old fibroblast population, either in quiescence or after a 24h-TGF-β-1 stimulation and (**H**) in the same conditions, mean quantification of immunofluorescence staining from young or old fibroblasts (Mean±SD, n=6); (**I**) Wound closure over 96h as measured by % of cell confluency for analysis of migration of young control fibroblasts (CT) or after transfection with siARP2/3 or with siScramble (Mean±SD, n=6); *p*-value *<0.05, **<0.01, ***<0.001. (**J**) Schematic representation of the actin dynamic polymerization between ARP2/3, CFL1, CORO1C regulators, adapted from *Mol Biol Cell. 2010; 21:3529-39*. Hypothetically, the low level of ARP2/3 in old cells might favor a potent deficiency in actin polymerization with as consequences, alteration of cell motility and capacity to interact with the ECM components. This could be reinforced by the low levels of CFL1. Moreover, the increase of CORO1C in old fibroblasts should induce a higher strength of the actin filaments, leading to cell rigidification with no renewal in the actin complex as a consequence of loss in cell contractility and effective migration.
